# Smoking and quitting inequalities among gender-diverse adults in Great Britain: a population-based study

**DOI:** 10.1186/s12889-026-27375-4

**Published:** 2026-04-17

**Authors:** Sharon Cox, Lion Shahab, Sarah Jackson

**Affiliations:** 1 Behavioural Research UK, Edinburgh, UK; 2 Department of Behavioural Science and Health, University College London, London, UK

**Keywords:** Gender-diversity, Tobacco, Inequalities, Inequity, Smoking

## Abstract

**Background:**

Tobacco smoking is a leading cause of health inequalities, and smoking rates are higher among people who are gender-diverse. This study examined smoking prevalence, dependence, quit attempts, and cessation behaviours among gender-diverse adults in Great Britain, compared with adults identifying as men or women.

**Methods:**

Cross-sectional data were drawn from the Smoking Toolkit Study, a nationally representative survey of adults aged 16 years and above in Great Britain, collected between October 2020 and June 2025. Gender identity was self-reported as male, female, or in another way, with the latter defined as gender-diverse. Outcomes included smoking prevalence, markers of tobacco addiction and motivation to quit, past-year quit attempts and motivations, quit success, and use of evidence-based cessation aids. Logistic and linear regression models were used to examine associations, adjusting for age and social grade and survey wave, with additional covariates included for quit success analyses.

**Results:**

The analytic sample consisted of 387,629 adults, including 906 (0.2%) individuals who identified as gender-diverse. Current tobacco smoking prevalence was higher among gender-diverse adults (28.0%) than among men (18.7%; aOR = 1.38, 95% CI 1.17–1.63) and women (14.8%; aOR = 1.76, 95% CI 1.49–2.07). Among current smokers, those who were gender-diverse were more likely to smoke within 30 min of waking compared with men (aOR = 1.45, 95% CI 1.08–1.94) and women (aOR = 1.52, 95% CI 1.13–2.04), indicating greater tobacco addiction. Motivation to quit was lower among gender-diverse smokers compared with women (aOR = 0.61, 95% CI 0.39–0.95) and possibly men (aOR = 0.67, 95% CI 0.43–1.04). Past-year quit attempts and quit success did not differ significantly by gender identity.

**Conclusions:**

Gender-diverse adults in Great Britain experience substantially higher smoking prevalence and greater tobacco addiction. While the quit attempt rates were broadly similar across groups, motivation to quit generally appeared lower among gender-diverse people who smoke. These findings highlight the need for inclusive, equity-focused tobacco control strategies that address the specific barriers faced by gender-diverse communities.

## Background

Tobacco smoking remains a leading cause of preventable death and disease globally [1–3] and is a key driver of health inequalities [1, 4, 5]. In Great Britain, while smoking rates have declined overall, these trends conceal persistent disparities [6]. Marginalised and minority groups—such as gender-diverse people—continue to experience disproportionately high rates of smoking [7, 8] and as a result, higher rates of tobacco-related cancers and cardiovascular illness [8, 9].

Gender diversity refers to an identity beyond a male and female binary. International evidence indicates that individuals who identify as gender-diverse are more likely to smoke, face greater challenges in quitting, and encounter structural and psychosocial barriers to cessation support [10–13]. These barriers are not confined to smoking cessation support but reflect barriers to health care and social support more generally [13, 14]. To achieve the UK’s ambition of a smoke-free population (≤ 5% smoking prevalence) by 2030 [15, 16] action must be inclusive and equity-focused, addressing the specific needs of priority groups, including gender-diverse communities [17].

While some systematic reviews and commentaries on gender diversity and smoking suggest that tailored approaches are more effective, [10, 12] the literature often conflates sexual orientation and gender identity, limiting the relevance and usefulness of the data for designing interventions [12]. In the UK, recent calls to improve the health of gender-diverse people have highlighted the need for public services—including tobacco control—to be inclusive and responsive [17]. However, large-scale evidence remains scarce. Many national datasets fail to include gender-diverse people within their analysis, often excluded due to small sample sizes, and over time, this leads to invisibility in research and policy.

This study addresses that gap by examining smoking and quitting behaviours among gender-diverse adults in Great Britain, comparing them to those who identify as male or female. To fully assess smoking disparities between genders, here we focus on smoking prevalence, quit attempts, cessation methods, as well as markers of addiction [18–21], and motivation to quit [22].

Using cross-sectional data from the Smoking Toolkit Study (STS), this study aims to examine the extent to which there are differences between gender-diverse adults and adults who identify as male and female in:

1. The proportion who report currently smoking (a) any tobacco, (b) cigarettes, and (c) exclusively non-cigarette tobacco;
2. Among people who currently smoke, the proportion who smoke within 30 min of waking, strength of urges to smoke, and cigarettes per day (as markers of tobacco addiction) and the proportion who report wanting to quit smoking in the next 3 months (as a marker of motivation);
3. The proportion of past-year smokers who report a past-year quit attempt, and among those, factors motivating the quit attempt;
4. The proportion of those who made a past-year quit attempt who report (a) continuous abstinence (i.e., quit success) and (b) use of evidence-based smoking cessation aids.

## Methods

### Pre-registration and ethical approval

The protocol for this study was pre-registered on the Open Science Framework: 10.17605/OSF.IO/2V5W3. Clinical trial number: not applicable.

Ethical approval is provided by the UCL Research Ethics Committee (0498/001). Participants provide informed consent to take part in the study, are treated in accordance with the Declaration of Helsinki, and all methods are carried out in accordance with relevant regulations. The data are not collected by UCL and are anonymised when received by UCL.

### Sample and recruitment

Data were drawn from the STS, a monthly cross-sectional survey of people (≥ 16 years) in Great Britain. The STS uses a hybrid sampling method combining random location and quota sampling, with data collected via telephone or online interviews; full details can be found elsewhere [23, 24]. Data were weighted with the rim (marginal) technique [25] (using the R survey package) to match the analytic sample to the proportions of the English population profile on the dimensions of age, social grade, region, housing tenure, ethnicity and working status within sex.

We included data from participants surveyed between October 2020, when data collection in Wales and Scotland started, and June 2025, the most recent data available at the time of analysis.

### Measures

#### Explanatory variable

Gender identity was assessed by self-report as male, female, and non-binary or my gender is not listed. Participants identifying as non-binary and my gender is not listed, the latter two are classified as “in another way” and here are defined as gender-diverse. Details on how this is classified by the organisation that collects the data can be found elsewhere [26].

### Outcomes

*Smoking status* was assessed by asking participants which of the following best applies to them:

- I smoke cigarettes (including hand-rolled) every day.
- I smoke cigarettes (including hand-rolled), but not every day.
- I do not smoke cigarettes at all, but I do smoke tobacco of some kind (e.g., pipe, cigar or shisha).
- I have stopped smoking completely in the last year.
- I stopped smoking completely more than a year ago.
- I have never been a smoker (i.e., smoked for a year or more).

Those indicating *a*,* b and c* were considered people who currently smoke tobacco. Those who respond *d* were considered people who have successfully quit smoking in the last year.

The following outcomes were examined:

1. Current smoking: In all adults, derived from the above question, we assessed current tobacco smoking (a-c), current cigarette smoking (a-b), exclusive non-cigarette tobacco smoking (c);
2. Smoking behaviour: In people who currently smoke: mean cigarettes per day (CPD), mean urges to smoke, the percentage who smoke within 30 min of waking, and the percentage who have high motivation to stop (‘really want and plan to stop within 3 months’) [18–22];
3. Quit attempts: In people who smoked in the past year, we assessed the percentage reporting a past-year quit attempt as measured by the question, ‘How many serious attempts to stop smoking have you made in the last 12 months? By serious attempt I mean you decided that you would try to make sure you never smoked again. Please include any attempt that you are currently making and please include any successful attempt made within the last year’.
  Among those reporting making one or more serious attempts to quit in the past year, the following outcome were assessed:
4. Motives for quitting: Motives for the most recent attempt to quit were assessed by the question: ‘Which of the following do you think contributed to you making the most recent attempt to quit?’

1. Advice from a GP/health professional
2. TV advert for a nicotine replacement product
3. Government TV/radio/press advert
4. Hearing about a new stop smoking treatment
5. A decision that smoking was too expensive
6. Being faced with smoking restrictions
7. I knew someone else who was stopping
8. Seeing a health warning on a cigarette packet
9. Being contacted by my local NHS Stop Smoking Services
10. Health problems I had at the time
11. A concern about future health problems
12. Attending a local stop smoking activity or event
13. Something said by family/friends/children
14. A significant birthday
15. Pregnancy
16. Just decided to quit
17. The coronavirus outbreak (from April 2020 on-wards)
18. Other (please specify)
  However, we only include for this study; (1) Advice from a GP/health professional; (5) A decision that smoking was too expensive; (7) I knew someone else who was stopping; (10) Health problems I had at the time; (11) A concern about future health problems; (13) Something said by family/friends/children, and; (15) Pregnancy. The decision to reduce responses was based on our previous studies (e.g. Jackson 2024, [27]) showing the most popular overall reasons for trying to stop smoking covered advice, cost, social factors, and health.
19. Quit success and methods: Among those who made a past-year quit attempt, we also present the percentage not currently smoking (i.e., quit success) and the percentage who used evidence-based cessation support (face-to-face behavioural support, prescription nicotine replacement therapy (NRT), electronic cigarettes (e-cigarettes) or prescription medication) dichotomised as yes/no.

### Covariates

Age range was categorised as: 16–24, 25–34, 35–44, 45–54, 55–64 and 65 + years. Social grade, measured using the National Readership Survey [28], is categorised as ‘AB’ (higher and intermediate managerial, administrative or professional managerial, administrative or professional), ‘C1’ (supervisory or clerical and junior managerial, administrative or professional), ‘C2’ (skilled manual workers), ‘D’ (semi- and unskilled manual workers) and ‘E’ (casual or lowest-grade workers, pensioners and others who depend on welfare). This was dichotomised into more advantaged (ABC1) and less advantaged (C2D1) groups. For the quit method and success, we also included alcohol use, as measured by the AUDIT-C [29], as a continuous variable. Smoking-related covariates included in this analysis also included the number of past-year attempts and the time since the quit attempt started. In a deviation from protocol, we include a survey wave covariate to account for seasonal variation, however formal trend analysis by gender identity was not conducted because the gender-diverse subgroup size within individual waves or annually was insufficient to produce stable wave-specific estimates.

### Analyses

Analyses were conducted in R (version 4.5.2). Data were weighted to represent GB population estimates (using srvyr and survey packages).

We present a descriptive table for all exposure (gender: gender-diverse, male and female) and outcome variables, reporting prevalence estimates with 95% CI for each group, or means and standard deviations for continuous variables. We provide inferential statistics using logistic regression to analyse associations (presenting odds ratios, OR) adjusting for key covariates, for all except motives which deviates from our protocol, but samples sizes were not large enough. To yield the magnitude of difference between those identifying as gender-diverse to male and also females we made these comparisons for each research question. To avoid over interpreting the data (i.e., ‘Table 2 fallacy’ [30]), we only reported the fully adjusted models, including the following covariates:

- Age, social grade and survey wave for analyses of RQs 1 through 3
- age, social grade, survey wave, strength of urges to smoke, use of evidence-based aids, alcohol use, number of past-year attempts and time since quit attempt started for RQ4 analysis

The gender-diverse subgroup (*n* = 906; 0.2% of the total sample) provided adequate sample sizes for the analyses of smoking prevalence and markers of addiction but resulted in limited precision for outcomes with smaller cell sizes, i.e., quit success, cessation aid use, and quit motives. For the latter, regression analyses were not conducted due to insufficient cell sizes and point estimates should be interpreted with appropriate caution. We report 95% confidence intervals in Table 1 to indicate any uncertainty around point estimates.

**Table 1:** Smoking characteristics and behaviours by gender identity

Variable	Men	Women	Gender-diverse	Adjusted Model: In Another Way vs. Men (ref)	Adjusted Model: In Another Way vs. Women (ref)
Sample Characteristics (Full Sample, *N* = 387,629)	*n* = 188,767	*n* = 197,545	*n* = 906		
Past-year smoking prevalence	22.1%	19.6%	29.0%	OR = 1.29 (1.10–1.51)	OR = 1.62 (1.38–1.90)
Current tobacco smoking prevalence	18.7% (17.4–19.1)	14.8% (14.4–15.1)	28.0% (24.8–31.1)	OR = 1.38 (1.17–1.63)	OR = 1.76 (1.49–2.07)
Current cigarette smoking prevalence	16.2% (15.8–16.5)	13.6% (13.2–13.9)	24.3% (21.3–27.3)	OR = 1.36 (1.14–1.61)	OR = 1.63 (1.37–1.93)
Exclusive non-cigarette tobacco smoking	2.2% (2.0-2.3)	1.2% (1.1–1.3)	3.6% (2.3–4.9)	OR = 1.27 (0.86–1.87)	OR = 2.30 (1.55–3.41)
Among Past-Year Smokers (*n* = 80,834)	*n* = 41,790	*n* = 38,704	*n* = 263		
Made past year quit attempt/s	33.7% (33.2–34.1)	37.4% (37.0-37.9)	32.7% (31.9–44.7)	OR = 1.04 (0.80–1.13)	OR = 0.88 (0.68–1.13)
Continuous abstinence (quit success)	25.5% (23.7–27.2)	26.3% (24.5–28.2)	22.2% (13.9–27.8)	OR = 0.99 (0.79–2.31)	OR = 0.96 (0.89–1.56)
Among Current Smokers (*n* = 74,341)					
Smoke within 30 min of waking	42.0% (40.8–43.3)	41.7% (40.3–43.0)	45.8% (38.9–52.7)	OR = 1.45 (1.08–1.94)	OR = 1.52 (1.13–2.04
Plan to quit in next 3 months	15.1% (14.2–16.0)	16.2% (15.2–17.2)	10.8% (6.6–15.0)	OR = 0.67 (0.43–1.04)	OR = 0.61 (0.39–0.95)
Strength of urges (0–5 scale), M (95% CI)	1.74 (1.71–1.77)	1.87 (1.84–1.90)	2.33 (2.10–2.55)	β = 0.68 (0.45–0.90)	β = 0.56 (0.33–0.79)
Cigarettes per day, M (95% CI)	10.8 (10.5–11.0)	9.15 (8.94–9.37)	13.7 (11.0-16.3)	β = 4.58 (1.92–7.24)	β = 6.47 (3.81–9.13)
Cessation Aids (Among Those Who Made Attempt) (*n* = 3617)					
Use of any evidence-based aid	5.5% (4.6–6.4)	6.9% (5.9-8.0)	1.1% (-1.1-3.3)	OR 0.88 (0.66- 0.97)	OR 0.72 (0.68–0.93)

Values are percentages with 95% confidence intervals in parentheses unless otherwise specified. M = Mean. OR = Odds Ratio, β = regression coefficient. Sample sizes vary by variable due to missing data. All analyses used weighted data and excluded observations with missing values for the specific variable being analysed. Regression models used complete case analysis, excluding observations with missing data on any covariate. RQ1-3 models (current smoking prevalence) adjusted for age and social grade. RQ4 model (continuous abstinence) adjusted for age, social grade, strength of urges, evidence-based aids (prescription medication, e-cigarettes, behavioural support), alcohol use, number of past-year attempts, and time since quit attempt started. “-” indicates no regression model was conducted for this variable

## Results

### Sample characteristics

A total of 387,629 adults were included in the analysis, comprising 188,767 men (48.7%), 197,545 women (51.0%), and 906 individuals who identified as gender-diverse (0.2%). This proportion is broadly consistent with, though slightly below, the 0.5% population estimates [30, 31].

In the total sample, 20.9% were past-year smokers, 19.2% were current tobacco smokers, and 18.4% were current cigarette smokers. Descriptive and adjusted results for smoking behaviours by gender identity are presented in Table 1.

### Smoking prevalence by gender

Gender-diverse adults had a higher prevalence across all measures of smoking. After adjustment for age and social grade, gender-diverse adults had significantly higher current tobacco smoking (28%) relative to men (18.7%: aOR = 1.38, 95% CI 1.17–1.63) and women (14.8%: aOR = 1.76, 95% CI 1.49–2.07). Patterns were consistent for past-year and current cigarette smoking. Exclusive non-cigarette tobacco use was relatively uncommon across all groups but was more prevalent among gender-diverse adults compared with women, with no clear difference relative to men.

### Markers of addiction and motivation among current smokers

Among current smokers, gender-diverse adults showed evidence of higher tobacco addiction: a greater proportion of gender-diverse smokers reported smoking within 30 min of waking compared with both men (aOR = 1.45, 95% CI 1.08–1.94) and women (aOR = 1.52, 95% CI 1.13–2.04).

Mean strength of urges to smoke was also highest among gender-diverse smokers. Adjusted regression analyses indicated significantly stronger urges among gender-diverse smokers relative to both men (β = 0.68, 95% CI 0.45–0.90) and women (β = 0.56, 95% CI 0.33–0.79). Gender-diverse smokers also reported significantly higher cigarette consumption, smoking on average 4.6 more cigarettes per day than men and 6.5 more than women, respectively.

In contrast, motivation to quit smoking was lower among gender-diverse smokers. After adjustment, the odds of planning to quit in the next 3 months were lower among gender-diverse smokers compared with women (aOR = 0.61, 95% CI 0.39–0.95, *p* = 0.028) and men (aOR = 0.67, 95% CI 0.43–1.04, *p* = 0.071), though it did not reach the significance threshold for the latter.

### Past-year quit attempts and motivating factors

Among past-year smokers, the proportion reporting at least one serious quit attempt in the previous 12 months was similar across gender groups; after adjusting for age and social grade, those who identified as gender-diverse did not differ from women (aOR = 0.88, 95% CI 0.68–1.13) or men (aOR = 1.04, 95% CI 0.80–1.34).

**Fig. 1:**
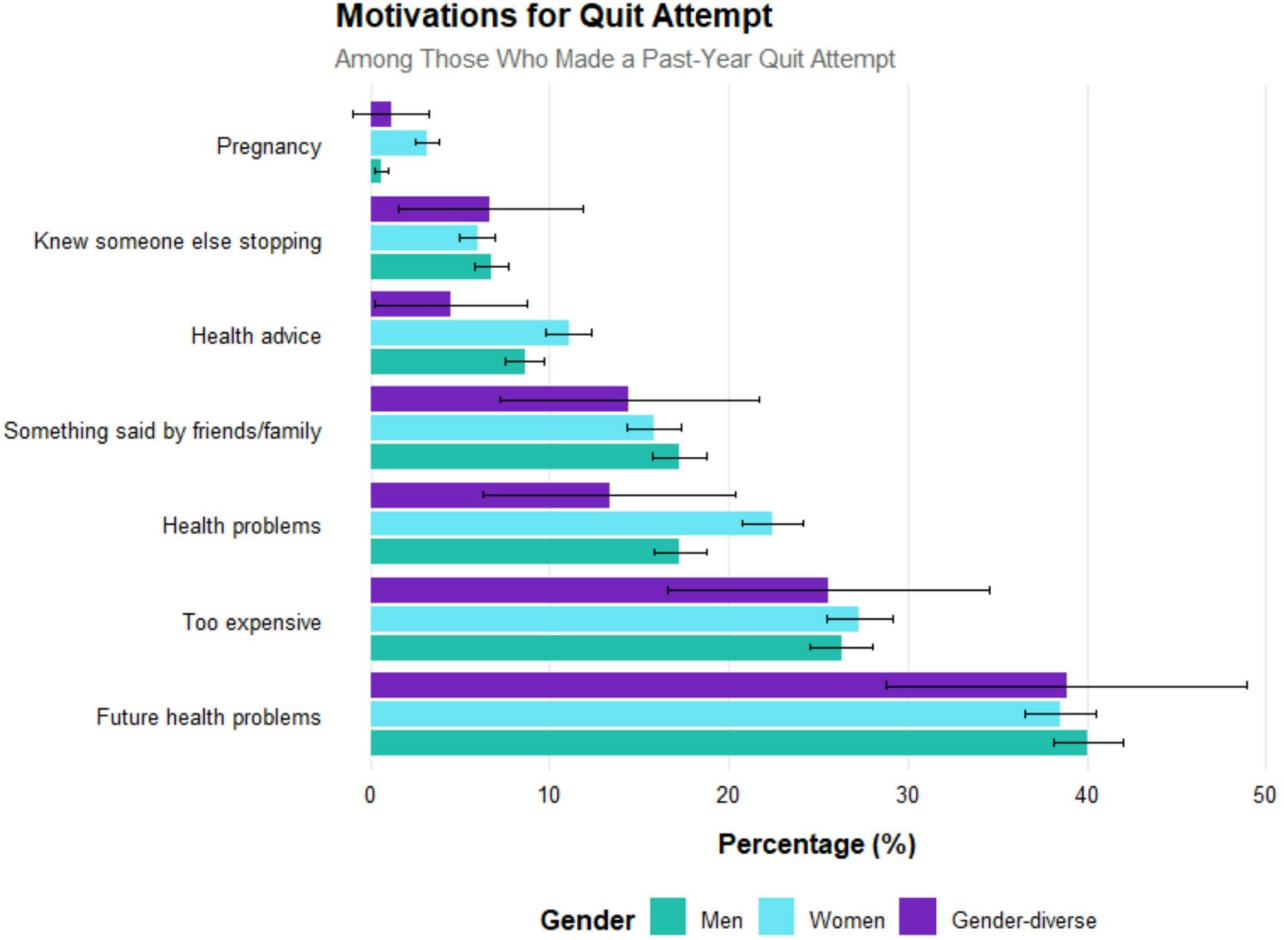
Among those reporting a past-year quit attempt, motives for the quit attempt by gender identity. Results show % and bar represent 95% confidence intervals

Distribution of specific motivating factors also appeared broadly similar across gender identities, see Fig. 1. Concern about future health problems was the most commonly reported motivation for a quit attempt, followed by the financial cost of smoking. However, formal regression analyses were not conducted for these outcomes due to limited sample size among gender-diverse respondents; and for both quit attempts and motives point estimates should be interpreted with caution given limited subgroup size.

### Quit success and use of evidence-based cessation aids

Among those who made a past-year quit attempt, continuous abstinence rates were comparable across groups. After adjustment, there was no evidence of a difference in quit success between gender-diverse adults and either men (aOR = 0.99, 95% CI 0.79–2.31) or women (aOR = 0.96, 95% CI 0.89–1.56).

Among those who had made a quit attempt, use of evidence-based cessation aids was lower among gender-diverse people (1.1%) compared with both men (5.5%; aOR 0.88, 95% CI 0.66–0.97) and women (6.9%; aOR 0.72, 95% CI 0.68–0.93). Again, owing to small sample sizes, point estimates should be interpreted with caution.

## Discussion

### Principal findings

This population-based study among adults in Great Britain highlights several smoking-related health inequalities experienced by gender-diverse individuals. After adjustment for key variables, gender-diverse adults demonstrated higher smoking prevalence, greater markers of tobacco addiction, and lower motivation to quit compared with both men and women. Our findings align with international evidence documenting elevated smoking rates among gender-diverse populations [10, 11, 32]. Given the cross-sectional design, the observed associations should not be interpreted as causal. The aim of this study was to describe the current status quo and to aid hypotheses generation only. The mechanistic explanations discussed below are offered as plausible hypotheses to contextualise the findings and guide future research.

The 28% current smoking prevalence among gender-diverse adults substantially exceeds rates observed in men (18%) and women (15%), representing a nearly 80% increased odds of smoking relative to women. Further, gender-diverse individuals showed multiple indicators of heavier, more dependent smoking patterns—including higher cigarette consumption, stronger urges to smoke, and greater likelihood of smoking soon after waking. These markers suggest potentially greater difficulty in achieving cessation [18–22]. Notably, while quit attempt rates were similar across groups, motivation to quit was significantly lower among gender-diverse smokers. However, the fact that quit success rates did not differ significantly by gender identity is an interesting finding and perhaps should be interpreted with caution given that we found use of evidence-based aids was also lower among gender-diverse people compared with men and women. The lower use of evidence-based cessation aids among gender-diverse adults who made a quit attempt (1.1%, compared with 5.5% among men and 6.9% among women) is a relevant finding for clinical action and policy, and qualitative research would be useful to further unpack the reasons for this. Several factors may contribute to this disparity. Gender-diverse individuals have reported negative healthcare experiences, a lack of provider knowledge about their needs, and a need to ‘go it alone’ which may discourage engagement with GP-based cessation pathways and prescription services [13, 33]. This is consistent with the patterns observed in Fig.  1, where advice from a GP or health professional appeared to be a less commonly cited motivator for quit attempts among gender-diverse respondents, though formal statistical comparison was not possible due to sample size constraints.

The elevated smoking prevalence and lower motivation to quit among gender-diverse adults likely reflect multiple intersecting factors. One plausible example is that of minority stress theory, which posits that stigma, discrimination, and social marginalisation can contribute to adverse health behaviours as coping mechanisms [34]. Specifically, gender-diverse individuals face persistent discrimination in healthcare, employment, and social settings, which may increase psychological distress and reliance on smoking as a stress management strategy (see Li et al., for a recent review) [32]. Other reasons, such as the feelings of general social exclusion but the sense of cultural identity and belonging smoking environments can afford should be seen as potentially reinforcing for why smoking continues and why motivation to give that up is low [13]. While these pressures may plausibly explain higher smoking rates, they also could explain lower motivation, unsuccessful attempts and accumulated dependence over time. Although, of course, people and their identities are simultaneously positioned across multiple categories [35], and for some, other intersecting factors will create and maintain barriers to cessation, these are outlined further in the limitations below.

### Implications for policy, practice and future research

These findings have important implications for tobacco control policy and practice in Great Britain. Achieving a smokefree future [15] will require targeted, equity-focused interventions that address the specific needs of gender-diverse communities. Current cessation services may not adequately address the barriers faced by this population. This includes avoiding gender-based assumptions, and acknowledging the specific stressors that gender-diverse people face [14].

Targeted interventions delivered in LGBTQIA+-affirming settings may be one route, and research from cancer prevention and screening studies suggests more needs to be understood on what ‘tailoring’ means and how this works in practice [36]. Other work evaluating community-based smoking cessation support is encouraging (e.g., The Last Drag) [33] but derives from the US and therefore has limited application to the UK context.

At a policy level, ensuring that gender identity is routinely collected in health surveys and monitoring systems is essential for tracking disparities and evaluating intervention effectiveness. The fact that only 0.2% of our sample identified as gender-diverse—slightly below population estimates of 0.5% [31] —underscores the importance of large sample sizes to enable robust analysis of health outcomes in this community.

Future work should examine the intersection of gender identity and sexual orientation as previous research shows that certain intersections of the two show higher smoking rates and face greater and uniquely different barriers to quitting [12, 37, 38]; meaning those identifying as gender-diverse should not be seen as a homogenous group.

Furthermore, future research should also examine alternative nicotine use patterns by gender identity as a means of assessing opportunities for interventions and prioritising need. It was beyond the original scope of this analysis to examine other forms of nicotine use, including non-combustible products (e.g., e-cigarettes and pouches) which may be used for either harm reduction by people who smoke or used as a new form of nicotine by those who have never smoked.

### Strengths and limitations

This study has several notable strengths. The large, nationally representative sample provides estimates of smoking behaviours across gender identities in Great Britain. The inclusion of multiple markers of tobacco addiction, motivation, and cessation behaviour provides a wider insight into smoking disparities. As noted below, there are limitations to how gender diversity is classified; nonetheless, it is important to highlight the inequalities and inequities experienced by minority groups which present with higher smoking rates.

However, several limitations should be acknowledged. The cross-sectional design precludes causal inference about mechanisms underlying the observed disparities. The measure of gender diversity is analysed here as individuals who identify “in another way,” which imperfect but is how the research team receives the data. Still, we appreciate that this does not capture the full spectrum of gender identities or allow for distinction between transgender, non-binary, and other gender-diverse identities. This broad categorisation may obscure important within-group variation. Sample sizes for gender-diverse adults, while sufficient for primary analyses, were limited for some secondary outcomes, particularly quit success and cessation aid use. The collapsing of data across the study period precluded examination of temporal trends in smoking behaviours. This is a notable limitation given the evolving healthcare landscape during the study window which had impacts on people’s smoking and nicotine use behaviours, including the COVID-19 pandemic [39] and population level shifts in tobacco and vaping usage patterns [40, 41]. Future analyses with larger pooled samples may enable trend analysis, complementing the cross-sectional findings presented here. Contemporary longitudinal research, such as the Queer Women’s Substance Use Over Time (QSOX) Project in Australia, highlights how substance use trajectories among gender and sexuality minorities are shaped by structural factors over time [13], and similar longitudinal work in the UK would be a valuable addition to this evidence base.

While the study adjusted for key demographic factors; important potential confounders were not available in the dataset. Sexual orientation, which is correlated with both gender-diverse identity and smoking behaviour [13, 37, 38], was not included in the models. Similarly, mental health conditions (including depression, anxiety, and minority stress exposures), experiences of discrimination and social exclusion, and markers of socioeconomic instability beyond social grade were unmeasured. These variables are plausibly associated with both gender identity and smoking outcomes, and their omission means that the adjusted odds ratios reported here may overestimate the independent contribution of gender identity per se to smoking disparities. Future studies should seek to include these variables to disentangle the pathways through which gender identity is associated with smoking behaviour.

Lastly, point estimates for outcomes, especially those with smaller cell sizes, should be interpreted with appropriate caution. However, while the small gender-diverse subgroup limits the precision of some estimates, we note that this study represents one of the largest population-based analyses of smoking behaviours among gender-diverse adults in the UK. The small subgroup size is itself a reflection of the underrepresentation of gender-diverse people in research, underscoring the importance of continued and routine data collection on gender identity in national health surveys.

## Conclusions

Gender-diverse adults in Great Britain experience a substantial and persistent smoking-related health inequity, characterised by higher smoking prevalence, greater tobacco addiction, and lower motivation to quit compared with men and women. These findings highlight the need for inclusive, equity-focused tobacco control strategies that recognise and address the specific barriers faced by gender-diverse communities.

## Data Availability

The data generated and analysed for this current study are available from the corresponding author on reasonable request.
